# Beyond fiber electronics

**DOI:** 10.1093/nsr/nwaf393

**Published:** 2025-09-17

**Authors:** Huisheng Peng

**Affiliations:** State Key Laboratory of Molecular Engineering of Polymers, Department of Macromolecular Science, and Institute of Fiber Materials and Devices, Fudan University, China

## Abstract

This perspective article presents some examples on how to figure out new ideas on doing science based on the author's own studies including fiber electronics.

The motivation for writing this perspective article originates from a sincere hope to present a pathway for young scientists—particularly junior faculty members, postdoctoral fellows and graduate students—to figure out important new ideas on doing research. It should be noted that, although I have been primarily regarded as a chemical researcher, my work extends far beyond the scope of chemistry. I will discuss my understanding on science simply along a timeline, rather than following a strict logic sequence, as the connections between these ideas are not always straightforward.

## FIBER ELECTRONICS

**Figure 1.**
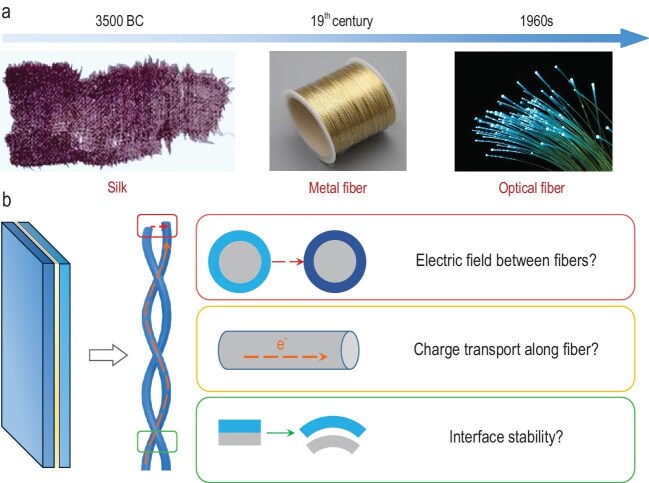
(a) Main events during the evolution of fiber materials. (b) Structure evolution of conventional planar to new fiber devices by changing from planar electrodes to fiber electrodes.

I started my independent research career along the discovery route from fiber materials to fiber devices >20 years ago. It is well known that fiber materials have played a critical role in pushing human civilization ahead (Fig. 1a). More than 5000 years ago, ancient Chinese people utilized natural fibers such as silk to weave textiles, marking a significant milestone in human civilization. During the nineteenth century, Heinrich Hertz and colleagues in the UK realized power transmission by inventing metal wires, advancing a revolution in the electrical industry. In the 1960s, Kuen Kao proposed the principle of light transmission through quartz fibers, opening up a new era of fiber-optical communication. With the further revolution in information technology, we clearly recognized that it was urgent to transform the above fiber materials into fiber devices that are flexible, elastic and miniaturized to satisfy booming new fields such as implantable and wearable electronics. They can also be woven into breathable electronic textiles, potentially changing our lives in the near future.

It was very difficult to develop fiber devices that worked as effectively as their planar counterparts. We had to face three main challenges when changing from planar electrodes to fiber electrodes for effective fiber devices (Fig. 1b). (i) How can a uniform electric field between fiber electrodes be achieved? (ii) How can charges move effectively along long fiber electrodes? (iii) How can a close and stable interface between the active material and the fiber electrode be maintained? We have solved these problems

mainly by synthesizing new materials and designing novel architectures, as summarized in several review articles [1–3]. To date, almost all electronic functions, including generating electricity, storing energy, emitting light, changing colors, computing, communicating and treating disease, have been realized through fiber devices [4–10]. We have further developed continuous fabrication methods that pave the way for large-scale production and application in industry [11]. With growing industrial efforts, some fiber devices are already available on the market, some have been produced on a large scale for next-generation products, and many others are under commercialization.

## METAL-BACKBONED POLYMER

About a decade ago, I began looking for new research directions. Polymers have played critical roles in human history due to their diverse and attractive properties. Natural polymers such as wool and cellulose have been used for tens of centuries. General polymers, including plastics and rubbers, have been synthesized to revolutionize the world in many directions in the past century. Both natural and general polymers typically have non-conjugated backbones, so they are generally insulating. Conducting polymers, often with conjugated backbones, were discovered more recently, expanding the scope for polymers. Of course, the backbones of all types of polymers are typically made of nonmetal atoms such as C, N, O and Si. Metal elements, which constitute >80% of the periodic table, have rarely been studied for use in polymer backbones.

**Figure 2.**
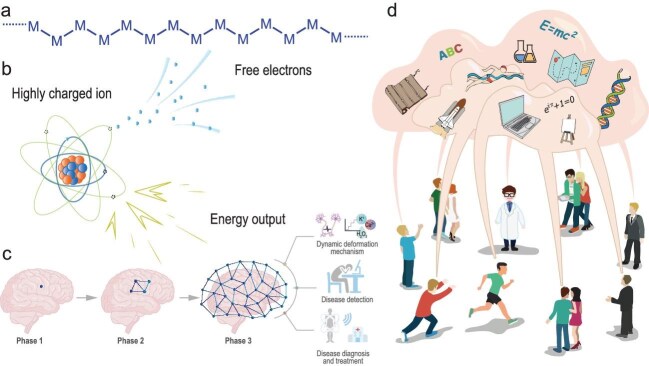
(a) Molecular structure of the backbone of metal-backboned polymers. Here, M represents a metal atom. Reprinted with permission from Ref. [12]. (b) Schematic of energy production through highly charged ions. Reprinted with permission from Ref. [17]. (c) Schematic of brain deformation mapping and promising applications. Reprinted with permission from Ref. [18]. (d) Schematic of transferring and sharing knowledge between electronic neurons. Reprinted with permission from Ref. [19].

An idea of making polymer backbones entirely out of metal atoms suddenly occurred to me while I was on a train back to Shanghai in 2017. After several years of hard work, we successfully synthesized a new type of polymers with backbones composed entirely of metal atoms and we called them metal-backboned polymers (Fig. 2a) [12–16]. In the typical synthesis process, multidentate scaffold ligands consisting of organic units act as an ideal 1D tunnel-like host to hold a single chain of metal atoms. Many types of metal atoms, including Ni, Pt, Cu and Co, have been chemically bonded to form metal-backboned polymers, which exhibit remarkable electronic and electrochemical characteristics, such as extremely high catalytic properties.

Although we have published some papers on the synthesis and properties of metal-backboned polymers, many challenges remain. For instance, how can metal atoms be chemically bonded stably? How can we scale up the synthesis for practical applications? How do electrons move along metal backbones? As it is very difficult to answer these questions with the current knowledge, we still have to face many critical problems in the design of metal-backboned polymers. Fortunately, metal-backboned polymers are now recognized as a promising new direction and important type of material that may revolutionize many fields, including chemistry, physics, energy science, biomedical science and electrical engineering. It is believed that this direction will advance rapidly through the joint efforts of multidisciplinary scientists.

## BREAKING IDEAS BEYOND CURRENT SCIENTIFIC LOGIC

Guided by a similar research philosophy, I continue to think about new ideas that may seem unbelievable or even ridiculous to other scientists. After careful consideration, I decide to share these ideas with the academic community, hoping to inspire more young scientists to step beyond conventional research paths.

### New energy devices

We are currently exploring entirely new energy devices with extremely high energy densities that far exceed those of the current counterparts. From the perspective of classic electrochemistry, both energy-harvesting and energy-converting devices typically utilize the outermost electrons, resulting in relatively low energy densities. For example, only one electron per lithium atom functions in electric transport. Classic lithium-ion batteries are approaching the theoretical limit of energy density for active materials. Although multivalent ions offer more electrons per ion, their energy densities remain unsatisfactory due to the rather low electron-number-to-atomic-mass ratio. In contrast, inner-shell electrons, which constitute a large proportion of atoms, remain untapped. The transition from excited to ground states in inner-shell electrons can release several orders of magnitude more energy, especially in heavy atoms.

I began to focus on this direction in late 2016, encountering a lot of failures. Eventually, we figured out a novel energy-generating system that utilizes highly charged ions. Energy was produced through the recombination of free electrons and highly charged ions, and was subsequently converted into electricity (Fig. 2b) [17]. These new energy devices have been verified by some interesting systems with the use of inner electrons, completely jumping out of the conventional thinking box. Although we recently published our first paper verifying the working mechanism, there is still a long way to go before practical energy devices—comparable to existing commercial systems—can be realized. It is necessary to effectively control inner-shell electron transitions and achieve material stability under extreme conditions for the realization of real energy devices.

### Dynamic mapping of whole brain

Monitoring the intrinsic properties of the brain is crucial for advancing the understanding of the brain. Among these properties, mechanical aspects, such as the real-time dynamic deformations of the brain tissue, have been relatively underexplored. However, mechanical properties are as critical as their electrical and chemical counterparts. For instance, in the heart, mechanical stress significantly influences electrical activity and overall cardiac health, while, in the auditory system, precise mechanical vibrations are essential for converting sound into neural signals. Similarly, in the brain, tissue deformations are not merely passive responses, but also actively contribute to various physiological and pathological processes. These deformations, for example, induce relative micromotions between neural devices and brain tissue, posing significant challenges in the longevity and reliability of chronic neural recordings. Moreover, brain deformations are implicated in developmental processes and diseases such as epilepsy and brain tumors. Exploring these mechanical properties offers a novel methodology for studying the brain and finding the missing puzzle pieces in brain science.

To this end, we spent several years developing implantable soft sensors to achieve real-time monitoring of brain deformation [18]. We began to dynamically map the brain deformation at a single point, then across an area, and finally across the whole brain (Fig. 2c). Throughout this process, we continuously improved the spatial resolution and monitoring dimensions, ultimately exploring complex mechanical interactions within the brain. We are currently integrating sensing technologies with advanced imaging and computational modeling technologies. This integration may create a dynamic, real-time model of brain deformation, thus providing an unprecedented level of detail about the mechanical environment of the brain. Such a model will be essential in understanding how brain deformations relate to neurological disorders, the efficacy of therapeutic interventions, and the performance of neural implants over time.

While the study of brain deformation is still in its early stage, it has the immense potential to fill a critical gap in brain science. This ongoing research aims to push the boundaries of what is possible in this field, ultimately contributing to a deeper understanding of brain function and improving outcomes in both clinical and research settings.

### A new learning paradigm for knowledge

The realization of brain mapping may further provide new opportunities to enhance human learning capability. From the emergence of animals on Earth to the evolution of humans over thousands of years, the typical learning model involves organisms receiving external information through the senses and undergoing repeated training to memorize and accumulate knowledge. The nature of knowledge can be mainly summarized as the generation of new synaptic connections and the formation of new neural activity patterns in neural circuits, which is time-consuming and requires extensive training. Even for human beings, the amount of knowledge acquired in a lifetime is very limited. Therefore, traditional learning methods are increasingly challenged by the explosive growth of knowledge and information.

Inspired by concepts in Wuxia (martial arts) novels that I read during middle school—in which knowledge can be transferred from an experienced swordsman to a newcomer in seconds to minutes with their palms against each other—I dreamed of developing a new learning pathway. To this end, we have proposed and verified a new learning paradigm of knowledge [19]. We implanted an electronic system, called an electronic brain, as the second brain of the human body. This electronic brain may consist of electronic neurons that mimic the functions of brain neurons. They can form new patterns of neural activity by creating new circuits between electronic and biological brains. Therefore, using cloud storage technology, the electronic brain can directly transfer knowledge from the cloud to humans without traditional training. Electronic neurons can efficiently establish or sever their interconnections when updating knowledge, avoiding the slow retraining process of biological neurons.

Of course, this technology still requires research and validation in both hardware and software. On the hardware side, single-cell precision neural electrodes for collection and stimulation still need further investigation. For instance, existing stimulation methods such as optogenetics and electrical stimulation are insufficient for achieving one-to-one stimulation between electronic and biological neurons. We are not able to form precise neural connections between the two. On the software side, real-time analysis and judgment algorithms for patterns of massive neural activities in the brain still need to be further improved. Among these, a scientific understanding of language and vision in the human neural network is particularly important. Additionally, the efficiency and flux of signal-processing ability remains to be enhanced, as the existing processing algorithms are inadequate for handling complex brain activities.

With advances in neuroscience and artificial intelligence, this new learning paradigm may become a general and effective strategy to transfer a large amount of knowledge from the cloud to humans and enable knowledge sharing between individuals (Fig. 2d). This could significantly enhance the learning efficiency and intelligence level of human beings.

### Fractional elements

According to the classic theory, the elements in the periodic table are organized by atomic number, which increases by integers. In other words, the next element has an atomic number exactly 1 greater than the previous one. This theory has guided the discovery of many new elements. Occasionally, while reading a science book, an unconventional idea occurred to me: could the difference in atomic numbers between two adjacent elements be <1? For instance, could it be 1/3, 2/3 or another non-integer value? This idea seems unbelievable and is also difficult to prove with the current knowledge.

Before Frederick Soddy proposed the isotope hypothesis, it was widely believed that the relative atomic mass of an element should be an integer multiple of the relative atomic mass of hydrogen (which equals 1)—this is known as ‘Prout’s hypothesis’. However, subsequent isotope studies discovered that, between element 82 (lead) and element 92 (uranium), there exist 40 atomic nuclei with different relative atomic masses, which contradicts the predictions of the periodic table. With the subsequent confirmation of isotopes, it becomes clear that isotopes are atoms of the same element with the same number of protons but different numbers of neutrons. As a neutron is 0.138% heavier than a proton, the relative atomic mass deviates from an integer as the number of neutrons increases. This demonstrated the limitations of the integer rule for atomic mass.

Inspired by this story, I speculate that non-integer elements might be possible, and I would like to call them ‘fractional elements’. If they exist, then many aspects of physical science could be rewritten, and numerous new materials could be thus discovered, potentially revolutionizing the world in unexpected ways.

To summarize, fiber electronics and metal-backboned polymers have gained recognition in both academia and industry. Studies on new energy devices, dynamic mapping of the brain, new learning paradigms of knowledge and fractional elements are still in their infancy. Nevertheless, I hope to emphasize that science requires bravery and imagination as a first step, followed by careful exploration and verification. It is thus essential for more researchers to follow these exciting new directions.

## References

[1] Zeng K, Shi X, Tang C et al. Nat Rev Mater 2023; 8: 552–61. 10.1038/s41578-023-00573-x

[2] Chen C, Feng J, Li J et al. Chem Rev 2023; 123: 613–62. 10.1021/acs.chemrev.2c00192 35977344

[3] Xu X, Xie S, Zhang Y et al. Angew Chem Int Ed 2019; 58: 13643–53. 10.1002/anie.201902425 30986329

[4] Ren J, Zhang Y, Bai W et al. Angew Chem Int Ed 2014; 53: 7864–9. 10.1002/anie.201402388 24899361

[5] Chen T, Wang S, Yang Z et al. Angew Chem Int Ed 2011; 50: 1815–9. 10.1002/anie.201003870 21328646

[6] Zhang Z, Guo K, Li Y et al. Nat Photon 2015; 9: 233–8. 10.1038/nphoton.2015.37

[7] Wang L, Xie S, Wang Z et al. Nat Biomed Eng 2020; 4: 159–71. 10.1038/s41551-019-0462-8 31659307

[8] Shi X, Zuo Y, Zhai P et al. Nature 2021; 591: 240–5. 10.1038/s41586-021-03295-8 33692559

[9] Ye L, Liao M, Zhang K et al. Nature 2024; 626: 313–8. 10.1038/s41586-023-06949-x 38326591

[10] Lu C, Jiang H, Cheng X et al. Nature 2024; 629: 86–91. 10.1038/s41586-024-07343-x 38658763

[11] He J, Lu C, Jiang H et al. Nature 2021; 597: 57–63. 10.1038/s41586-021-03772-0 34471277

[12] Zeng K, Peng H. Chin J Polym Sci 2023; 41: 3–6. 10.1007/s10118-022-2887-x

[13] Zeng K, Yang Y, Xu J et al. Angew Chem Int Ed 2023; 62: e202216060. 10.1002/anie.202216060 36640110

[14] Wang N, Zeng K, Zheng Y et al. Angew Chem Int Ed 2024; 63: e202403415. 10.1002/anie.202403415 38573437

[15] Zhang Y, Zeng K, Yang Y et al. Acta Polym Sin 2023; 54: 413–7.

[16] Zhang Y, Zeng K, Wu Y et al. Sci Bull 2025; 68: 105–9.

[17] Jiang Y, Zeng K, Yang Z et al. Natl Sci Rev 2025; 12: nwaf139. 10.1093/nsr/nwaf139 40353187 PMC12063093

[18] Liu Z, Tang C, Li J et al. Adv Electron Mater 2024; 10: 2300732. 10.1002/aelm.202300732

[19] Wang Z, Wang J, Shi X et al. Adv Healthcare Mater 2023; 12: 2203247. 10.1002/adhm.202203247

